# Two Rare Pathogenic *HBB* Variants in a Patient with β-Thalassemia Intermedia

**DOI:** 10.4274/tjh.galenos.2020.2020.0020

**Published:** 2020-05-06

**Authors:** Veysel Sabri Hançer, Tunç Fışgın, Murat Büyükdoğan

**Affiliations:** 1 İstinye University Faculty of Medicine, Department of Medical Biology, İstanbul, Turkey; 2 Altınbaş University Faculty of Medicine, Department of Pediatrics, İstanbul, Turkey; 3 İstinye University Faculty of Medicine, Department of Medical Genetics, İstanbul, Turkey

**Keywords:** Beta thalassemia, HBB, Variation

## To the Editor,

**Figure 1:**
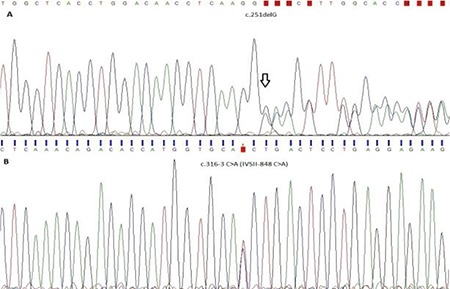
Electropherograms of the patient.

The β-thalassemias are a group of hereditary disorders with autosomal recessive inheritance characterized by the presence of defective synthesis of the β-globin chain, an integral component of the hemoglobin molecule, resulting in either partial synthesis (β^+^) or complete absence (β^0^) [1]. The disease reaches a high frequency in the Mediterranean Basin, Africa, the Middle East, the Indian subcontinent, and Southeast Asia [2]. According to the World Health Organization, the frequency of abnormal hemoglobin is 7% globally [3]. β-Thalassemia major is characterized by completely inhibited synthesis of beta chains [4], and so it must be treated, generally by transfusion therapy [4]. The β-thalassemia major phenotype has homozygotes or compound heterozygotes for β^0^ or β^+^ genes. Generally, mutations targeting the coding regions of the gene and conservative regions on the exon-intron boundary lead to β^0^-thalassemia, and mutations in regions that do not encode β^+^-thalassemia. In contrast to the major type, the presence of one normal gene in heterozygotes usually leads to enough normal β-globin chain synthesis so that the affected individuals are usually asymptomatic with only hypochromic and microcytic red blood cells. This condition is referred to as β-thalassemia minor [5]. β-Thalassemia intermedia clinically differs from the major and minor ones with respect to the necessity of transfusion. The degree of anemia for β-thalassemia major is more aggravated than that for β-thalassemia intermedia. The genotype of β-thalassemia intermedia is mostly homozygous or compound heterozygous [5]. A 14-year-old male Iraqi patient with Turkish origins presented with infection, mild hepatomegaly, and loss of appetite. Laboratory findings were as follows: white blood cell count, 13.53x10^9^/L; red blood cell count, 3.84x10^12^/L; platelet count, 367x10^9^/L; hemoglobin, 7.7 g/dL; hematocrit, 26.3%; mean corpuscular hemoglobin, 22.7 pg; and mean corpuscular volume, 68.5 fL. The patient had no transfusion history. Written informed consent was obtained. A peripheral blood sample was collected in an EDTA-containing tube. Genomic DNA was extracted from the white blood cells. The *HBB* gene was amplified as 2 polymerase chain reaction (PCR) fragments (from the -101 position to the Poly-A signal) using 40 ng of genomic DNA in reaction volumes of 25 µL. After PCR amplification, sequencing was performed using the BigDye Terminator v3.1 Cycle Sequencing Kit. The patient had heterozygous c.251delG (p.Gly84fs, rs193922555, β^0^) and heterozygous c.316-3 C>A (IVSII-848 C>A, rs33913413, β^+^) pathologic variants, as shown in Figure 1. Sequencing analysis showed that the father had heterozygous c.251delG and the mother had heterozygous c.316-3 C>A variants. The global frequency of c.251delG and c.316-3 C>A is unknown and 0.00002%, respectively [6]. c.316-3 C>A is observed at a frequency of 0.4% in Turkey [7] and 2.9% in Iraq [8]. c.251delG is observed at 0.2% in Turkey [9] and 10.1% in northern Iraq [10].

These findings may be useful for genetic counseling, premarital/prenatal diagnosis, and prevention programs.
